# Descriptive Study of Damage Caused by the Rhinoceros Beetle, *Oryctes agamemnon*, and Its Influence on Date Palm Oases of Rjim Maatoug, Tunisia

**DOI:** 10.1673/031.008.5701

**Published:** 2008-10-08

**Authors:** Rasmi Soltani, Chaieb lkbel, Med Habib Ben Hamouda

**Affiliations:** ^1^ Department of Biological Sciences and Plant Protection, Agronomics Sciences Institute of Chott Meriam 4042 Sousse, Tunisia

**Keywords:** Scarabaeidae, offshoots, oviposition, palm tree collapse

## Abstract

*Oryctes agamemnon* (Burmeister 1847) (Coleoptera, Scarabaeidae) was accidentally introduced in the southwestern oases of Tunisia (Tozeur) around 1980 and spread to the Rjim Maatoug region. In these areas *O. agamemnon* was specific to date palm trees causing severe damage that can result in potential danger due to collapse of the tree. This study was conducted from April 2004 to March 2006 in 4 sites in the region of Rjim Maatoug. Different levels of palm tree attack were determined, ovioposition sites were identified, and pest damage was described in detail to specify their relative importance and to indicate factors governing palm tree attack. Eggs were individually oviposited in the attacked parts. Dead parts of palm trees were the main target of *O. agamemnon* including the respiratory roots, tough, trunk bark, dry petiole and the periphery of the crown. The crown itself was not attacked. Feeding by larvae caused significant damage. The biggest danger occurred when heavy attacks of larvae invaded the respiratory roots at the level of the soil, and secondarily on the periphery of the crown, which can result in fungal diseases. Several cases of Deglet Nour date palm tree collapse were caused by this pest in Rjim Maatoug. Attacks on other parts of the tree were without danger for the palm tree. In the absence of pest management, application of a quarantine program combined with field cultivation techniques could help farmers significantly decrease attack of *O. agamemnon* on palm trees.

## Introduction

In southwestern region of Tunisia, the date palm is often prone to phytophagous attacks caused by various insects, in particular in the zones of high date production of Djerid (Gouvernorat of Tozeur), and Nefzaoua (Gouvernorat of Kebili). The three principal species threatening date production are the carob moth, *Ectomyeloïs ceratoniae* (Zeller 1839), the white scale, *Parlatoria blanchardi* (Targioni Tozzetti 1892), the acarina Boufaroua, *Oligonychus afrasiaticus* (McGregor 1939), and a new pest, the beetle *Oryctes agamemnon* (Burmeister 1847) (Coleoptera, Scarabaeidae). The latter is an exotic pest originating from the Middle East (Baraud 1985). It was introduced accidentally for the first time in the late 1970s to the early 1980's into the palm plantations of Mrah lahouar in Tozeur. It came from the countries of the Golf region and was disseminated by offshoots of the variety Deglet Nour to the oases of Ibn Chabbat of the same area (Anonymous 2000). By the middle 1980's *O. agamemnon* had reached the Rjim Maatoug zone after new oases were installed using biological material from infested oases of Tozeur.

The gradual acclimatization of *O. agamemnon* to Tunisian oases conditions was simultaneously accompanied by a change in the date palms hosts. The Deglet Nour variety, which is the most cultivated within these oases, became its principal target. Consequently, it has became an important pest of date palm trees. Currently, the most significant pest danger has occurred in certain oases of Tozeur and Kebili states. Although it has not had any direct effect on production, it directly affects some sensitive parts of the date palm tree. Thus after many years of infestation, attacked parts can become weak threatening the sudden fall of the whole palm (Soltani 2004).

In the absence of biological and/or chemical control methods, *O. agamemnon* became acclimatized, and increased its population size to the point that it became difficult to control. The problem was discovered at the end of 1990s, but by this time they had become uncontrollable. In spite of the danger that *O. agamemnon* is likely to present for the future of date palm culture in the region, it has been neglected and no serious study has been devoted to it (Soltani 2004).

The objectives of this study were to do a detailed examination of damage, determine larval feeding behaviour, adult oviposition sites, dispersal methods, and effects on palm trees in the oases of Rjim Maatoug. Methods that could be used to prevent dispersion were also examined.

## Materials and Methods

### Study site

Observations started at the beginning of April 2004 in the area of Rjim Maatoug located in Kebili governorate, 120 km in the south-western area of this city, just south of Chott El Djerid. This area was characterized by a continental Saharan climate, an average rainfall lower than 100 mm/year, an average temperature of 21°C with extremes of 55°C in the shade in summer and 7°C in winter, sandy ground and desert vegetation limited mainly to the species *Ephedra alata alenda* (Stapf), *Spartidium saharae* (Cosson and Durieu) and date palm trees within oases.

Currently, the zone comprises 5 oases in production (Rjim Maatoug, Ferdaous 1 and 2 and Nasr 1 and 2) covering a total surface of 1440 hectares where observations took place. Oases in this area are continental and of the modern type with a mean planting space of 9 × 9 m. The principal vegetation was date palm trees with a marked predominance of the Deglet Nour variety and very few market-gardening or fodder practices for farmer consumption.

### Localization of oviposition and breeding sites in nature

Determination of oviposition sites was carried out while looking for various instars of the insect. This was done three times per week during the summer, for three years from 2004 to 2007. Eggs were collected from different oviposition sites of the palm tree. Breeding sites were determined using the same methodology but was done weekly during the entire year.

### Damage localization, symptoms and damage description

A general survey of all sites of the region was done to identify the principal existent categories of oases, the repartition of the species within it and also to determine attacked parts of palm tree where different instars of the *O. agamemnon* lived. Sampling for the second part was realized in a systematic way, during the entire period of the study, by choosing palm trees within the oases situated at each corner and in the middle. Samples were composed of hundreds of palm trees.

Simultaneously, exterior examination of all invaded parts was done in different sites of the region to determine all symptoms of attack by this pest. Damage description was based on meticulous observations of hundreds date palm trees and offshoots between 2004 and 2007, including percent infestation, and distribution of the attack.

## Results

### Organization of the date palm Root system

Being a monocotyledon, the date palm has no tap root. Its root system is fasciculated and roots are fibrous, similar to a maize plant. The date palm root system is divided into four zones (Oihabi 1991; Zaid et al. 2002):

*Zone I*, the respiratory zone, the respiratory zone is the base of the palm no more than 25 cm depth and laterally a maximum of 0.5 m away from the stipe. The roots in this zone are mainly primary and secondary type with a respiratory role.

*Zone II*, the nutritional zone, is large with the highest proportion of primary and secondary roots. These roots are between 0.90 and 1.50 m depth and project laterally beyond the tree's canopy.

*Zone III*, the absorbing zone, is usually found at a depth of 1.5 to 1.8 m. These primary roots are present with decreasing density from top to bottom.

*Zone IV*, when the underground water is deep, roots extended down into this zone.

### Trunk

The trunks of the date palm, also called stem or stipe, were vertical, cylindrical and columnar of the same girth all the way up. They were brown in colour, lignified and without any ramification. The average circumference was from 1 to 1.10 m. The trunk was composed of tough, fibrous vascular bundles cemented together in a matrix of cellular tissue that was lignified near the outer part of the trunk. The trunk was covered for several years with the bases of the old dry fronds, making it rough, but with age these bases weather and the trunk became smoother with visible cicatrization of the bases.

### Leaves

Depending on the variety, age of the palm and the environmental conditions, leaves of a date palm were 3 to 6 m long (4 m average) and had a normal life of 3 to 7 years. The greatest width of the frond midrib attains 0.5 m, but elsewhere it was only half this size and rapidly narrowed from the base upwards. The frond midrib or petiole was relatively triangular in cross section with two lateral angles and one dorsal. It was bare of spines for a short distance but full of spines on both sides thereafter. Intermediate zones have spine-like leaflets, also called leaflet-like spines.

### Damage localization and symptoms

Larvae constituted the harmful stage of *O. agamemnon* to palm tree in Tunisian oases. Larvae were never seen outside of the attacked parts of the palm tree. The adult insect was the only visible stage during the summer period. Also, damage was not externally visible on the attacked parts.

Larval instars were nourished on secondary parts, with little effect on the date palm tree. In the oases of the region, palm tree attacks were localised mainly at the base, particularly at the superficial part of the root, known as respiratory part and secondary on higher trunk levels including different components of the dry petiole levels and the periphery of the crown (tough, dry petiole, stem bark and fronds base of the crown). It should be noted that initial damage to the tree was localised as a result of the attack sites of the female beetle in obscure sites that were wet and well protected.

Regular surveys enabled a detailed description of damage to determine favourable conditions for pest attack. The attack of *O. agamemnon* on the palm tree causing symptoms of damage that are very specific and can pass unperceived during the first years of invasion because larvae live in obscure and well protected places between hairy or fibrous roots and tough layers protected by the dry petiole that can contain larvae in the interior tender wood.

**Figure 1.**
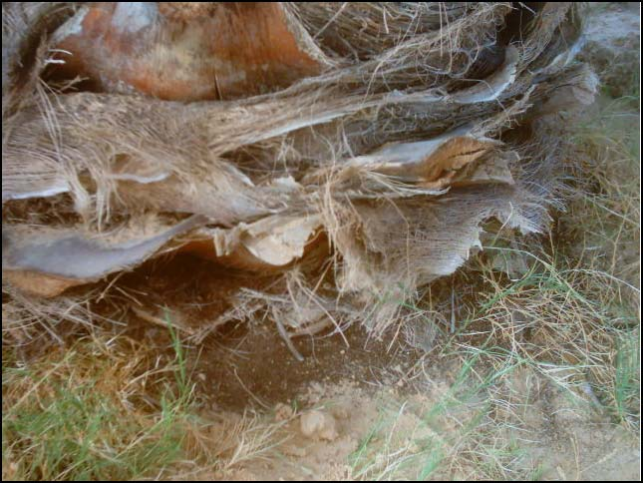
Powder on the ground showing symptoms of attack

Symptoms of attack differed according to their position. On the respiratory roots, two cases were identified: i) In recent invaded roots, it was too difficult to determine the plant situation, infested or not, at least during the two first years of invasion. Exterior evidence of infestation depends mainly on the size of the larval population. So, in a recently invaded palm tree it is difficult to detect the early presence of attack because the amount of damage is small compared to the entirely roots volume, ii) In old attacked palm trees, attacks were detectable by the existence of a brown powder similar to mature compost, what was scattered on the ground at the base next to the side of the palm tree and sometimes visible on higher levels of the tough surrounding the frond midrib (Figure 1). The existence of this powder showed that the tree had been attacked. Entry sites (holes) where adults oviposited, or where adults leave the tree after eclosion were evident. However, symptoms of attack on the higher parts of stem were sometimes visible on the dry petioles at the end of first generation. It appeared in the form of a brown powder scattered on superficial and visible tough layers surrounding the frond midrib of under the attacked level. The exterior symptoms at this level depend on the size of the existing population between the dry petiole and the stem bark. When the number of larvae exceeded three larvae it was possible to detect the presence of *O. agamemnon* due to larvae moving between two neighbouring frond midribs possibly because of lack of food. Another indicator of a high level attack on stems is the ease of which the dry petiole and tough can be pulled up as it becomes friable at its base. This has not been reported previously.

In contrast to productive palm trees, infested offshoots showed visible external symptoms especially in plantations after being planted separately from the tree. Symptoms were seen by a partial drying of the side of attacked part that can lead to a slower or arrest of development. During development, attacked young green palms became deformed with a wrinkled and weak aspect. They eventually turn yellow, desiccate, and die. However, attacked offshoots linked to mother plant remain alive and continue their development normally. There are no important visible symptoms because larvae are displaced superficially on offshoots and dig deeply into the hairy roots of the mother plant.

### Attack on the respiratory roots

The respiratory roots, previously described, were subdivided into two parts, an aerial part (or collet) situated in the top of the ground and an underground basal part (Figure 1). In the studied palm plantations, the aerial root showed the following characteristics which varied depending on the plant age and development level: measured height between the surface of the ground and the first stage of frond midrib was from 0 to 80 cm with a maximum diameter of 80 cm. An exterior fibrous, desiccated and hardened peripheral part was distinguished in contact with the ambient air. Its thickness was less than 5 cm, without any food value for the beetle and was not attacked. Its role is to protect the second internal part.

The internal part of the respiratory roots were voluminous and composed of fibrous (hairy) roots that were white in colour, wet, tender, non-woody and with high food value. This part provides sufficient food and better protection for several instars and constitutes the principal environment for embryonic and post-embryonic development.

After burrowing between fibrous roots, the female begins to oviposit. Eggs in palm trees attacked for the first time were deposited individually either directly between fine hairy roots, or in coarse granules chewed and grouped together by female inside an agglomeration of hairy roots. In trees that were damaged by previous attacks, oviposition primarily occurred in the decayed substrate of roots that that constitute the result of damage during previous years (crumbs that fall during larval feeding). Eggs were grouped in limited areas but were deposited singly in a random way and separate by a fine substrate layer.

Following hatching of the eggs, each larva digs a gallery where moulting and metamorphosis occurred. Different larval instars, especially the second and third instars, caused damage that appeared in the form of non-aligned lateral galleries or tunnels in hairy roots. These excavated tunnels were filled with a coating of liquid faecal material that may provides protection for the larvae. Field observations showed that different palm tree tissues were progressively devastated as larvae move. This phenomenon was described as feeding displacement (Soltani 2004). Repeated observations in various oases confirmed this hypothesis and also showed the general invasion of the respiratory root by larvae. There was no preferential orientation of attack.

**Figure 2.**
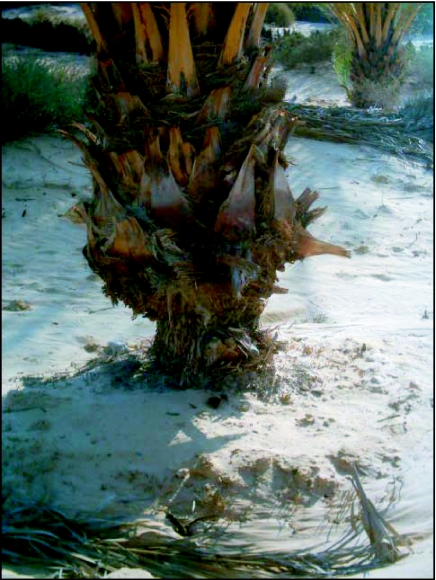
Aerial respiratory roots damage

Larval crowding in a confined area, especially of third instar, can lead to an overlapping and an interpenetration of these tunnels that results in the formation of larger holes from which larvae tend to dig deeper galleries. This phenomenon, repeated over several years, constitute a potential danger to the whole palm tree and can result in its base becoming unbalanced by the weakening of its basal support, and as a consequence, increasing the risk of its collapse in response to the least amount of wind (Figure 2). Horizontal and epicentric destruction of the perimeter of the fibrous roots can reach the central zone represented by the conducting vessels.

The danger of collapse increased considerably when the aerial roots were hidden under sand, which was a specific and a very frequent phenomenon in Rjim Maatoug oases (but rarely occured in the Djerid oases) owing to the abundance of wind-blown sandy that caused burial of these roots especially when they were shallow. Under these conditions, the humidity of buried roots was maintained favourable by the 12-day cycle of irrigation which resulted in the lateral development of the aerial roots whose fibres became very abundant. Thus the wind blown sand contributed to creation of conditions favourable to survival of larvae and considerably decreased the risk of palm tree collapse by increasing the tree anchorage to soil.

Attack on the underground respiratory roots, located below the aerial roots, induced weakening of the anchorage of the tree to the ground owing to partial or entire destruction of the lateral fibrous roots that hold it to the ground. The initial stages of attack were similar to the preceding except that access to this zone was done using either direct or indirect methods of entry.

There are two ways that adults have direct access to the basal part. 1) A female can use either an older existing hole or dig a new entry hole at the base of aerial roots in contact with soil. 2) Alternatively, they can use offshoots at the base of the palm tree that make the basal root zone easily accessible to larvae as their anchoring position to the mother plant provides direct access to its under-ground part. In this case, females preferred to deposit their eggs deeply around the linked space with the mother plant. After hatching, larvae fed superficially on offshoot tissues and on the fibrous roots of the mother plant. During the three years of observations, no case of entrance occurred inside offshoots when it was still connected to the mother plant; most of the larvae continued their development on the tissue of the mother plant, only two to three larvae of third instar developed on the superficial parts of the offshoots (tough, bark stem).

Adults were particularly attracted to unclean palm trees, or, to the Deglet Nour variety that was characterized by abundant offshoots at the base of the tree that can exceed fifteen per tree, a phenomenon that can considerably amplify the existing larval population on roots. After pulling up offshoots, females were still attracted to the older part of offshoots linked to the mother plant and most of the deposited eggs were concentrated around it.

It was clear from these results that the number of offshoots per palm tree base contributed to adult attraction and to an increase in the number of larvae in respiratory roots. This phenomenon increased the damage, and over times it reduces anchoring of palm tree to the soil and increases its risk of collapse. Also, it was evident that insects were more attracted to parts of the tree that had been injured.

A secondary route of entry can take place when attack was located on the lower half of the aerial root, with the galleries oriented downward. This phenomenon was only recorded in the event of overpopulation and food insufficiency in the lower aerial root. Attack by this second route of entry was rare and only observed in case of male trees.

The existence of a significant larval population in the underground respiratory roots can result in a generalized attack of its perimeter. This phenomenon was usually observed in the male palm trees in the oases of the area. As a direct result of this attack when larval residues were totally eliminated, conducting vessels were exposed to the ambient air (Figure 2).

Laying eggs in the sand between lateral underground fibrous roots constituted another direct access of the larvae to the basal aerial roots. In the Rjim Maatoug Development Office oasis this was a new phenomenon especially observed on buried respiratory roots of unkempt male palm trees, rarely on Deglet Nour variety and absent for common varieties. In the lateral areas around the trees, when samples are taken away from the principal underground respiratory roots, the number of larvae decrease in a proportional way with the decrease in concentration of the fibrous roots. The summer and the middle of autumn are the best for development and feeding activity. Eggs, and first and second instars constitute the principal populations observed at a depth of 15 to 20 cm feeding between fibrous roots in soil. But in winter, the unfavourable conditions cause the third larval instar, that are the main component of population, to migrate to the principal underground respiratory roots. Only a few of these larvae remain in the superficial layers of soil, 10 to 15 cm deep in the lateral areas. On maintained male palm trees the existing population of larvae were observed all around the perimeter and divided into a main group observed on the principal underground respiratory roots and a secondary group localized laterally at a distance reaching 40 cm from the tree and 20 cm deep in the ground. At this level, massive section and consumption of lateral roots by large populations can weaken of the lateral palm tree's anchor to the ground. Populations per palm tree in different sites, without considering the variety and the sex, varied between 1 and 137 larvae with the maximum found on male palm trees and the minimum on common variety. Comparison between these two groups shows that the population was mainly localised on the aerial part for Deglet Nour variety and on the fibrous roots in the male palms with the largest numbers in the underground roots.

From these results it is evident that palm tree survival is controlled by three parameters, the quantity of food available from the roots, the yearly population of larvae present and time in years that increases the possibility of repetitive attacks.

**Figure 3.**
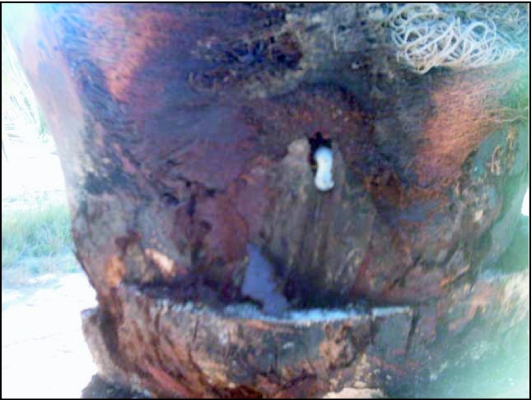
Larvae damaging tough and stem bark

### Attack on the stem periphery

The stem extends between the first stage of dry petiole just below the aerial roots and the crown periphery. Attacks were located under dry petiole stages and stem bark on tough, stem barks and dry petioles which were made up of dead tissues. The follow-up of damage caused in the Rjim Maatoug Development Office oasis clearly showed invasion of different parts of the stem. In all cases of the Deglet Nour variety females laid eggs between layers of tough after preparation of an area for egg laying formed from agglomeration of substrate granules. They were almost cylindrical in shape (length exceed sometimes 10 cm, diameter less than 1.5 cm) made in arbitrarily locations in tough layers. The number of deposited eggs varied from one to six and depended on the position of the invaded level. After hatching, new larvae fed directly on substrate granules which were easy to chew and later they move to tough tissues. Damage due to feeding by first and second instars larvae was minor due to the short duration of these instars and their small size. They fed mainly on tough until the second moult occurs. However, second instars larvae can attack the external bark stem surface and dry petiole, which was visible as superficial wounds.

Third instar larvae have robust mouth parts and initially attacked tough causing significant damage and creating varied geometrical forms of galleries in its layers (Figure 3). However, when groups are present in a limited space the amount of tough was insufficient to nourish these larvae during their long developmental period and consequently attacked stem bark, dry petiole and/or tough of the midrib of fronds. Larvae dug galleries on tough between the two neighbouring frond midribs. This phenomenon was frequent on the higher level of the stem situated under the crown.

**Figure 4.**
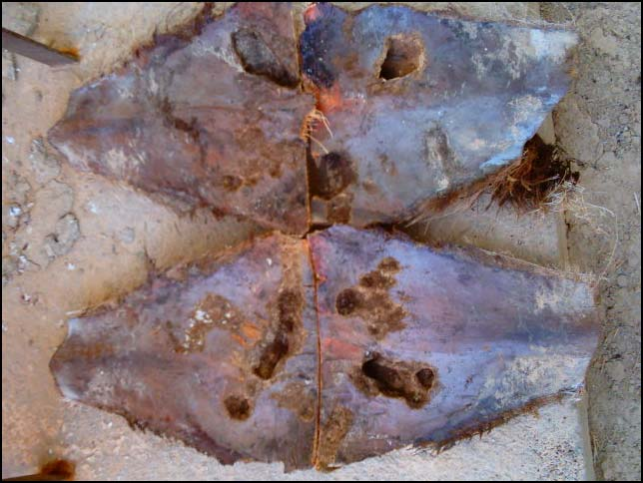
Dry petiole showing larval hole penetration and damage

Stem damage was superficial, not reaching 2 cm of depth, but can be 10 centimetres laterally when the attack on this level was long. Larvae were never seen inside the trunk. Generally, dry petiole was the target of third instar larvae. With their strong buccal mouthparts they are able to perforate its external hard surface and penetrate inside to feed on its tender soft wood. Larva passed the remainder of their lifecycle in the interior of dry petiole. Pupation occurred at the end of the tunnel from which the adult emerged. Attack on dry petiole was either superficial and limited to external cuts or invasion when there was passage to the inside (Figure 4). The tunnels dug at various levels of attack (roots, dry petiole) were standard for larvae of the same instars and in all cases their diameter exceeded slightly the diameter of larva. At the end of the feeding cycle, dry petiole was almost empty of its consumable contents, which accounted for 1/3 to 2/5 of its total volume, depending on how long the larva fed. Larvae situated on high exterior parts of the stem were covered and protected by a thick layer of compost, remnants of attacked parts, and by the dry petiole.

Quantification of the number of larvae feeding on dry petiole was measured according to its position compared to the crown. It revealed large variability ranging between 0 and 5 larvae per dry petiole base. The attack rate rose towards the crown. Generally, when measured from the base to the top of the stem the number of deposited eggs increased proportionally with food availability and quality which was mainly influenced by the humidity. The data revealed one larva per dry petiole base on the lower level; 1 to 3 larvae per dry petiole base were found on the middle part of stems and 2 to 5 larvae per dry petiole base on the last six petiole stage located just below the crown. A well developed dry petiole can support a maximum of two larvae of the third instar, which was rarely observed on dry petiole of male trees, but the most frequent case is one larva per dry petiole whereas the remainder fed on tough and stem bark when they move below the dry petiole. Generally, the number of larvae varied with regard to the volume and food quantity available to larvae from dry petiole. Also, a dry petiole level is not completely invaded by larvae because some of them, and their lower parts, are completely avoided. Larvae destroy tough, tender wood of bark and dry petiole only to feed. Comparison of the feeding tunnels compared to the length of stay confirmed this hypothesis. Normally a given dry petiole of the variety Deglet Nour was attacked only once, i.e., no repeated attack occurred on the layer of tough and dry petiole which received attacks previously.

The exception was observed only on one vigorous male palm tree on the oasis of the Rjim Maatoug Development Office oasis, where many cases of repeated attacks occurred on the basal levels of dry petioles in contact with the higher part of respiratory roots. In this case, an average of 3.66 eggs were counted in an average volume of 11 cm^3^ of the firm material.

In the Rjim Maatoug oases, stem attacks were rare but generalized in the oasis of the Rjim Maatoug Development Office oasis and in other small oases that are well managed and maintained. The number of collected larvae varied from 8 to 83 larvae on the Deglet Nour variety, from zero to 29 larvae on the basal level of male trees and were almost absent on the common variety. The variation in number on the variety Deglet Nour was dependent on the height of the stem which ranged from 1 to 4 meters.

Contrary to previous cases, in the Ferdaous and Nasr oases, stem attacks were very rare and limited only to the two first dry petiole stages situated right above the roots and which constituted an elongation of hairy roots parts i.e. a formation of hairy roots that was observed under the dry petioles that result in a higher extension of the respiratory roots. The absence of attacks throughout stem was caused by environmental conditions especially air moisture which was influenced by lack of shade due to the weak development of the of palm trees and to the larger space between them (9 × 9 meters) and to the spaced frequencies of irrigation in the region.

### The crown periphery

Attack on the periphery or at the base of the crown was mainly observed in most palm trees of the Rjim Maatoug Development Office oasis especially on the four oldest stages of green palms. In the other oases the presence of larvae was observed in rare cases in well-maintained parcels. Larvae were present in the crown periphery of green palms on the tender and wet tough and on stem bark. Green palms bases are usually never attacked because of the hardness of the wood that is still alive and gorged with water, and field observations showed that oviposition activity rarely occurred in the crown periphery. Then, how to explain the presence of larvae? The fact that larval population increased towards the crown and its density was highest at the top of the stem was discussed above. So, in presence of an overpopulation of larvae, they can move either behind the dry petiole or to the base of the crown. This was confirmed by the presence of tunnels between two successive levels at the extremity of green palms.

Larvae on the crown periphery can be the origin of indirect damage that is the result of excessive moisture combined with the high summer temperatures leading to harmful cryptogamic diseases that always occur after trunk invasion.

### Offshoots

Offshoots were present in tufts around the base of palm tree and this was a characteristic of uncleaned palm tree which constituted the more searched and targeted place for adult attack. The offshoots were targeted by larvae because of their soft wood.

Invasion of offshoots takes place as follows. Females deposited eggs directly on the offshoot's tough or beside it on the mother plant. After hatching, first and second instars larvae fed where the eggs were laid, as well as on the bark of offshoots. Attacks on the heart of offshoot were mainly caused by the third larval instar that have strong mandibles making it able to destroy the external hard wood. However, it is important to note that the heart was never invaded when the offshoot was still linked to mother plant. In this case, larvae of third instar either ate the external parts of offshoots or the mother plant tissues. This was confirmed by examining 447 offshoots from different sites of the region; no cases of attack on the heart occurred. All larvae of the third instar, that varied in number between one and three per offshoot, were present on superficial tissues where they continued their development after the offshoots were planted. Frequently, only one larva penetrated inside the heart part of offshoots, usually, by its base or sometimes laterally, then larva fed on tender wood, where it lived until pupation. The rest of larvae (one to two) continued their development on tough and bark stem of offshoots. Usually, an offshoot ensures the development of only one larva.

**Figure 5.**
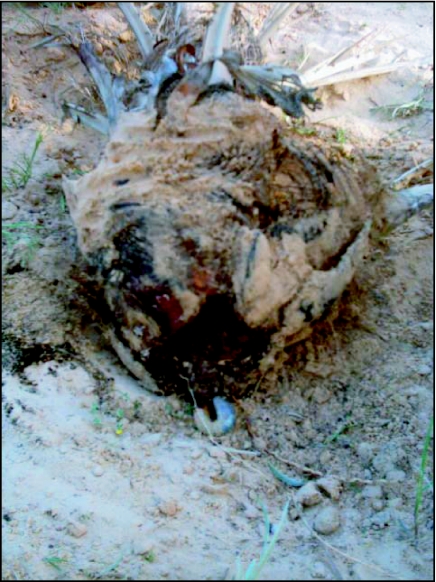
Offshoots attack and damage

Offshoots are used for oases extension and installation. In the Rjim Maatoug zone, the percentage of offshoot attack reached 100% in heavily infested palm plantations. Attacked offshoots showed a partial drying located on the side of the attacked part that can lead to a deceleration or arrest of development. At the end of this process, mortality of offshoots was caused by total drying, especially after separation from the mother plants. When planted, offshoots are autonomous and the drying process was accelerated. Rot installation due to high moisture generated by larvae presence can also occur (Figure 5).

Observation of offshoots after being pulled up revealed that the causes of drying and mortality were due to destruction of the roots or to partial or total consumption of the internal part (heart) comprising the sap conducting vessels. These cases were very frequent in palm plantations that lack replacement and where the percentage of success was low.

### Means of prevention

Currently, in absence of any effective means for control (by natural, biological or chemical means) of *O. Agamemnon* in infested oases of Tunisia, and given the potential danger that this species can represent for the palm plantations it is necessary to exploit conditions required by this species to control it or decrease its populations and its impact. The interventions to be applied are minimal and more for preventive purposes than curative. The solutions suggested include collection of adults and larvae of *O. agamemnon* using traps, the application of manure and reducing the number of offshoots per palm tree and their incineration.

Collection of adults and larvae is the only solution currently used by the Agriculture Development Regional Commissariat services. It requires significant specialized human resources and consequently a significant investment to carry out this task. The duration of collections is rather long, sometimes exceeding a whole season to cover all the infested area (approximately 1150 ha). Although this method contributes to the reduction of insect's population, it presents certain disadvantages such as the absence of specialized manpower and the meticulous work involved. Its application must be done before the reproductive period of *O. agamemnon* and, in the past, this basic point was not respected since it was applied during the summer season.

In order to avoid creation of new habitats and to decrease the propagation of *O. agamemnon*, we must have information relative to its spread, means of dispersion and application of a severe quarantine programme. Initially it is necessary to target the principal dispersion means of the species. One way to achieve this would be to prohibit the movement of offshoots from habitat sources. For example, clearing offshoots tufts from the base of the palm tree leaving only those necessary (a maximum of 4 per palm tree) as reserves for replacement of trees within oases. The unused offshoots must be burned. The effectiveness of this solution can be increased by collaborating with extension services to develop a serious campaign against the multiplication and dispersion of *O. agamemnon* that will make farmers aware of the subject. By imposing such a procedure in all infested zones, infestation will be reduced in all of the oases and damage from this insect will be reduced.

## Discussion

The attack of palm tree in Rjim Maatoug is different from other palm plantations of the Djerid (Tozeur) area. In Rjim Maatoug oviposition occurs without preference for orientation on the tree, whereas in the other plantations it was concentrated mainly on the north side of different attacked parts (Soltani 2004). A possible explanation for this phenomenon is the existence of a considerable population of this pest in the Rjim Maatoug oases that resulted in the massive and repeated attacks over several years that considerably reduced the available food quantity for *O. agamemnon*, which could have led to the change of behaviour during oviposition activity. A second hypothesis is that food resources became limited and exhausted, particularly in root parts. A new phenomenon that could explain the beginning of exhaustion of food resources in this level is the lateral attack of the respiratory roots, especially, on pollinisator.

In the oases of Ferdaous and Nasr the aerial components of the stem (tough and dry petioles) cannot ensure completion of the full biological cycle of the beetle. This was presumably due to different environmental conditions within these oases especially humidity which is low in plantations during the summer, the season of adult activities. This parameter was influenced by occurrence of shade, space between palm trees (which is important) and irrigation using a 12-day frequency cycle. Under dry conditions the aerial part of the stem with dry petiole and tough cannot ensure completion of the full biological cycle of the beetle. Thus the lack of stem attack was generated by unfavourable conditions for the development of *O. agamemnon* in the oases. Med Saeed (2004) confirmed that *O. agamemnon was* a root borer and was not a stem borer in the United Arab Emirates.

Usually taller palms were less attacked than younger lower palms (silhouette effect). This hypothesis was advanced by Soltani (2004) in the infested oases of Tozeur (Tunisia) and was confirmed in the well-maintained oasis of Rjim Maatoug. Young palm tree wood is soft, and more easily attacked by *O. agamemnon* than old palm trees. In contrast *Oryctes rhinoceros*, which attacks coconut palms, prefers to attack the older, taller trees (Bedford 1980; Sivapragassam 2003).

Thus, differences in attack between the Rjim Maatoug Development Office oasis and other oases of the region is mainly due to palm development which was controlled by water availability and the space between palm trees. The crown periphery attacks were closely linked to water availability controlling palm tree vigour and palm development.

Our results concerning the heart attacks of the palm tree are contradicted by those of Khoualdia et al. (1997) who mentioned the attack of these parts by this pest contradicted in part, the results of Soltani (2004) who was not sure about this phenomenon in the Djerid oases. In this work and to explain the absence of heart palms attack, we found that when going higher on the crown, the palms were more tightened which made the heart part inaccessible and restricted access of beetles. For green palms attacks, our results agree with results cited by Soltani (2004) in the Djerid oases but contradict the results obtained in the same region, by Khoualdia et al. (1997) and Med Saeed (2004) referring to green palms attacks which were superficially injured.

The failure of offshoots in plantations was mainly due to the non-existence of effective control methods. Chemical treatment of offshoots before plantation may be effective at the present moment as existing larvae were killed, but this is a temporary solution as new infestation is possible in future generations.

Large differences in behaviour are evident in the similar species, *O. rhinoceros* in pacific coconut plantations. First, contrary to *O. agamemnon* that causes damage mainly during the larval stage, damage and crop loss of coconut palms by *O. rhinoceros* is only caused by the adult beetle. The adult beetle burrows into the crown of the palm and feeds internally on the soft developing fronds before they have unfolded. When the damaged fronds unfold they show large angular cuts in the sides, while sometimes the end of the frond is completely cut off. The consequent loss of the leaf area may affect the palms adversely. Light attacks may have no discernible effect, but heavy attacks can kill the palm. Intermediate attacks weaken the palm and reduce nut production (Sivapragasam 2003). Kouassi et *al*. (2006) mentioned that *Oryctes monoceros* is the most serious pest in coconut plantations, causing up to 40% damage in tropical Africa, especially in the Ivory Coast. Damage is generally caused by adults making feeding galleries in the apical section of young palms, but also of mature palms when populations are large. Larvae develop in decaying dead palm wood.

Control of the Scarabaeidae, and especially of the *Oryctes* species, was above all preventive. Control becomes very difficult when breeding places are not removed (Ohler 1999). Waterhouse (1987) stated that the special difficulties were encountered for the control of the rhinoceros beetle because of the nature of the pest. A traditional approach to coconut rhinoceros beetle control, practiced in the Pacific, was for climbers to use barbed spears to extract the beetles from their feeding tunnels. This technique prevents the beetle from inflicting damage on other palms (Gressit 1953 cited in Waterhouse et al. 1987). For countries that still free from infestation, a severe quarantine program must be practiced to prevent the introduction of these species. The handpicking of larvae was also another method which can reduce effectively the existing population (Soltani 2004)

In Asia and Africa considerable numbers of natural enemies have been listed as attacking *O. rhinoceros* and other species of the genus with somewhat similar habits, example: *Scolia ruficormis, Platymeris laevicollis* and others. However, biological control has also been successfully used during the last half of the 20^th^ century especially against *O. rhinoceros* in the Pacific (Waterhouse et al. 1987). The fungi *Metharizium anispoliae* and *Baculovirus oryctes* were described since 1912 but they were not exploited (Friederiches 1920; Ramie et al. 1999). The dispersion of this fungus was successfully used for the genus *Oryctes* and especially with the species *O. rhinoceros* where the rate of infestation can reach more than 50%. When Huger (1966) discovered the *Rhabdionvirus oryctes* (also called *Baculvirus oryctes*) in Malaysia, research on arthropod and fungi natural enemies ceased. The efficiency of this virus in field varied from 38 % in Tanzania (Seguni et al. 1999), 84% in Tonga, between 57 and 68% in Fiji (Bedford 1980) and = 43% in Western Samoa.

## Conclusions

Species introduction via import of offshoots for new plantations permit larvae to survive and to continue their biological cycle under favourable conditions. The offshoots provided protection against all types of biotic and abiotic external aggressions. All these factors combined with the ignorance of the species supported its introduction into the new Saharan conditions of Rjim Maatoug.

The ignorance of *Oryctes* damage, ignorance of the species and confusion of its larval instars with other similar species of white grubs, in addition to the absence of chemical and biological control, allowed the continuous exponential increase in the population of *O. agamemnon*. This situation will not cease increasing as long as there is no serious intervention to limit the existing populations and to avoid the appearance of new infestations spreading to other oases.

The damage caused by *O. agamemnon* attacks is invisible during first years of invasion since they take place in protected and well-hidden parts of date palm tree (roots, tough, dry petiole, green palm base). Symptoms will be visible outside only at very advanced stages of the attack by the appearance of the food waste scattered on the ground at the palm tree base and on the dry petiole. These symptoms are visible only when the attack has reached the critical state that could result in the sudden and abrupt fall of the tree.

Larvae cause serious damage. They move between destroyed tissues to feed and for this reason we describe this movement as food displacement. The real danger lies in root attack that constitutes the basic support for the date palm. But, this danger relates to number of larvae and the food quantity available in the respiratory roots. In fact, light attacks may have no discernible effect, but heavy attacks can lead to sudden collapse of the palm. Attack on the stem is without notable danger to the palm tree since all the targeted parts have died and are not important for the vital functions of the plant.

Crown attack becomes dangerous if it results in infestation by fungal diseases that are supported by excessive moisture and high temperature. In all cases, this damage has no influence on the date palm tree productivity so they are without economic importance during the period passed between the first generation infestation by *O. agamemnon* and collapse of the palm tree. Finally, it is possible to qualify *O. agamemnon* as a cancer of palm trees since it destroys tissues slowly without effect, but causes abrupt death of the palm tree by its fall.
